# THE IDA (INTERFERENCE WITH DAILY ACTIVITIES) SCALE: A NEW MEASURE OF FUNCTION IN MILD COGNITIVE IMPAIRMENT

**DOI:** 10.1093/geroni/igae098.2983

**Published:** 2024-12-31

**Authors:** Ruth Tappen, Robin Jarvis-Powers, David Newman, Mónica Rosselli, Joshua Conniff, KwangSoo Yang, Jinwoo Jang, Ashlee Li

**Affiliations:** Florida Atlantic University, Boca Raton, Florida, United States; St. Mary’s Medical Center, West Palm Beach, Florida, United States; Florida Atlantic University, Boca Raton, Florida, United States; Florida Atlantic University, Boca Raton, Florida, United States; Florida Atlantic University, Boca Raton, Florida, United States; Florida Atlantic University, Boca Raton, Florida, United States; Florida Atlantic University, Boca Raton, Florida, United States; Florida Atlantic University, Boca Raton, Florida, United States

## Abstract

The functional decrements associated with mild cognitive impairment (MCI) are subtle, difficult to detect and often not reported by patients, yet may interfere with daily activities and be associated with patient concern, sometimes distress. Development of the IDA (Interference with Daily Activities) Scale began with a review of Memory Center patient records and interviews of 53 individuals who attended cognitive screenings sponsored by a second state-supported Memory Disorder Center. It was noted that participants reported few decrements when asked directly but endorsed far more when presented with a list of possible changes. Of 125 original items developed, 85 were retained for further testing based upon the interview results, participant endorsement of the item or supporting literature. They were then tested in two community samples, first reducing them to 65 items and then to 28 on the basis of significant (p≤.05) differences between those with MCI and those who were unimpaired and frequency of endorsement by those with MCI. The final version contains 22 items related directly to cognitive function and 6 items related to the psychosocial effects of the functional difficulties encountered. Internal consistency (Cronbach’s alpha) was.87, test-retest reliability over three months was.81, and criterion validity in comparison with the ECog scale was excellent (r=.72). IRT analysis indicated good fit and item discrimination. Use of the IDA Scale can contribute to a more comprehensive picture of the individual patient’s condition and current situation in clinical and research contexts.

